# Evaluation of the Possibility of Identifying a Complex Polygonal Tram Track Layout Using Multiple Satellite Measurements

**DOI:** 10.3390/s20164408

**Published:** 2020-08-07

**Authors:** Andrzej Wilk, Cezary Specht, Wladyslaw Koc, Krzysztof Karwowski, Jacek Skibicki, Jacek Szmagliński, Piotr Chrostowski, Pawel Dabrowski, Mariusz Specht, Marek Zienkiewicz, Slawomir Judek, Marcin Skóra, Sławomir Grulkowski

**Affiliations:** 1 Department of Electrified Transportation, Gdańsk University of Technology, Gabriela Narutowicza 11-12, 80-233 Gdańsk, Poland; andrzej.wilk@pg.edu.pl (A.W.); wladyslaw.koc@pg.edu.pl (W.K.); krzysztof.karwowski@pg.edu.pl (K.K.); jacek.skibicki@pg.edu.pl (J.S.); slawomir.judek@pg.edu.pl (S.J.); 2 Department of Geodesy and Oceanography, Gdynia Maritime University, Morska 81-87, 81-225 Gdynia, Poland; c.specht@wn.am.gdynia.pl (C.S.); p.dabrowski@wn.am.gdynia.pl (P.D.); 3 Department of Rail Transportation and Bridges, Gdańsk University of Technology, Gabriela Narutowicza 11-12, 80-233 Gdańsk, Poland; jacek.szmaglinski@pg.edu.pl (J.S.); slawomir.grulkowski@pg.edu.pl (S.G.); 4 Department of Transport and Logistics, Gdynia Maritime University, Morska 81-87, 81-225 Gdynia, Poland; m.specht@wn.am.gdynia.pl; 5 Department of Geodesy, Gdańsk University of Technology, Gabriela Narutowicza 11-12, 80-233 Gdańsk, Poland; marek.zienkiewicz@pg.edu.pl; 6 Department of Navigation and Marine Hydrography, Polish Naval Academy, Inżyniera Jana Śmidowicza 69, 81-103 Gdynia, Poland; m.skora@amw.gdynia.pl

**Keywords:** geometric layout of railway track, track axis identification, tramway loop, route’s polygon, GNSS mobile measurements

## Abstract

We present the main assumptions about the algorithmization of the analysis of measurement data recorded in mobile satellite measurements. The research team from the Gdańsk University of Technology and the Maritime University in Gdynia, as part of a research project conducted in cooperation with PKP PLK (Polish Railway Infrastructure Manager), developed algorithms supporting the identification and assessment of track axis layout. This article presents selected issues concerning the identification of a tramway line’s axis system. For this purpose, the supporting algorithm was developed and measurement data recorded using Global Navigation Satellite System (GNSS) techniques was evaluated and analyzed. The discussed algorithm identifies main track directions from multi-device data and repeated position recordings. In order to observe the influence of crucial factors, the investigated route was carefully selected. The chosen tramway track was characterized by its location in various field conditions and a diversified and complex geometric layout. The analysis of the obtained results was focused on the assessment of the signal’s dispersion and repeatability using residuals in relation to the estimated track’s direction. The presented methodology is intended to support railway infrastructure management processes, mainly in planning and maintenance through an efficient inventory of the infrastructure in service.

## 1. Introduction

In recent years, the managers of rail infrastructure have been increasingly recreating the course of railroads, as part of the overall inventory, in the global coordinate system. Initially, the geometric system in the global respect—the concept of Absolute Track Geometry (ATG)—was applied only to selected lines, which were characterized by specific operating conditions, such as railway lines of high operational priority (high speed rail lines) or sections coexisting with other structures of transport infrastructure, such as bridges, tunnels or squares. Other lines were measured using conventional measurement methods [1,2]. This was due to the relatively large labor input associated with the measurement and reproduction of the coordinates of the track courses in the global systems of spatial references. At present, in the processes of the modernization of railway lines, railway geodetic control networks are mounted more and more often, which is related to the state of geodetic networks. They allow the effective reconstruction of the track axis in the global system by tacheometric measurement (backward cutting to several reference points).

The real revolution in the field of track axis reconstruction was brought about by mobile surveying methods, which allow a continuous measurement at a higher speed than in the case of static or quasi static tacheometry. Due to techniques such as Global Navigation Satellite System (GNSS), Inertial Navigation System (INS) and Mobile Laser Scanning (MLS), the effective inventory of all lines at the railway network scale is now possible [3,4,5,6,7,8]. Moreover, these techniques enable a mobile measurement of coordinates with a high density of data recording at a relatively high speed of the measurement process. In extreme cases, it can even be the measurement with the speed of regular trains [9]. Contemporary methods of data processing are also worth mentioning. With frequencies of data recording of tens of hertz, it is necessary to use efficient algorithms for processing the measurement signal. The signal achieved is the so-called point cloud, which without further interpretation does not contribute valuable information. Hence, the signals should be interpreted in such a way that the data set can be converted into the model representation of geometric systems of the track, mainly in the horizontal plane (straight sections, transition curves and circular arcs), and also in the vertical alignment (sections of a constant slope and sections in vertical arcs) [10,11,12]. Because of the large number of collected coordinates, the effective analysis must include methods of automatic selection of particular sections of geometric systems [13,14,15,16].

Until recently, a questionable issue was the accuracy of GNSS measurements. The methods using tacheometers and railway control networks guarantee an accuracy of up to several millimeters. On the other hand, until recently, such accuracy in GNSS measurements was difficult to achieve. At present, it has been reported that an accuracy of about a 1 cm level is achievable also in this survey technique. Generally, the positions along the track are obtained based on the conversion of measurement signals from the different devices, based on the data fusion algorithms [17,18]. Furthermore, the accuracy often analyzed relates to certain specific measurement conditions, but railroads’ conditions are varied. 

For more than 10 years, the authors have been developing the technique of satellite measurements in order to evaluate implementation possibilities of this method [19,20]. At present, as part of the project InnoSatTrack shared with the Polish Railways, current possibilities of using GNSS, INS and MLS systems are subject to assessment for the purposes of track axis inventory (position and shape) [21,22] as well as for train positioning possibilities [23]. In the works under way, the biggest accent is put on GNSS technology, because the positioning of devices with high accuracy is the key to reconstruct the track axis in the global coordinates system [24]. In turn, measurements using additional devices, such as marine satellite compasses, inertial devices or laser scanners perform auxiliary functions, in particular for the purpose of determining corrections on the basis of additional information about the movement of measurement platform relative to the track [25,26].

In this paper we present the assessment of the possibility of determining the main directions of a polygon system, based on the example of a complex geometric system of the track. The main directions of the route are defined as tangents to horizontal curves in their boundaries with straight sections. It could be assumed that in specific cases the main direction covers a straight track section or a tangent between reverse curves. According to the above definition, the polygon of the route is understood as a system of main directions defined in the Cartesian coordinate system.

Geometric layouts in railway lines are usually characterized by mild changes of curvature (long straight lines and transition curves, large radii). For this reason, the authors tested both the measurement methods and data analysis methodology in relation to layouts of more diverse geometry. The examples of such systems are railway lines of agglomeration importance and mainly tramway lines, which in conditions of urban development require the use of short geometric sections [27,28]. In such situations, the exact reconstruction of the route’s polygon is a prerequisite for the proper reconstruction of the system of non-linear sections by using analytical methods of design [29,30,31,32].

An additional advantage of the analysis of the system on the tramway network is a varied degree of the occurrence of field screens. In such a situation the assessment of the accuracy of the reconstruction of the polygon is more reliable and indicates the potential possibilities for planning measurement campaigns on the lines of rail transport.

## 2. Materials and Methods

### 2.1. Characteristics of the Measured Tramway Route

For the analysis of the main directions, a tramway line including a street terminus in the district of Gdańsk Brzeźno (coastal town in northern Poland) was selected. Choosing the route, the following assumptions were taken into account:

- The route should be characterized by diversified visibility conditions of GNSS satellites, in order to verify and assess the impact of the number of visible satellites (their distribution on the horizon) on the possibility of the reconstruction of track geometry;
- The measuring section should have a diversified condition of track (structural and geometrical) so as to assess the impact of the state on the reconstruction quality;
- The measuring section should allow multiple measurements, in order to assess the reproducibility of the results obtained related to the parameters of the identified geometric system of the axis of track.

**Figure 1:**
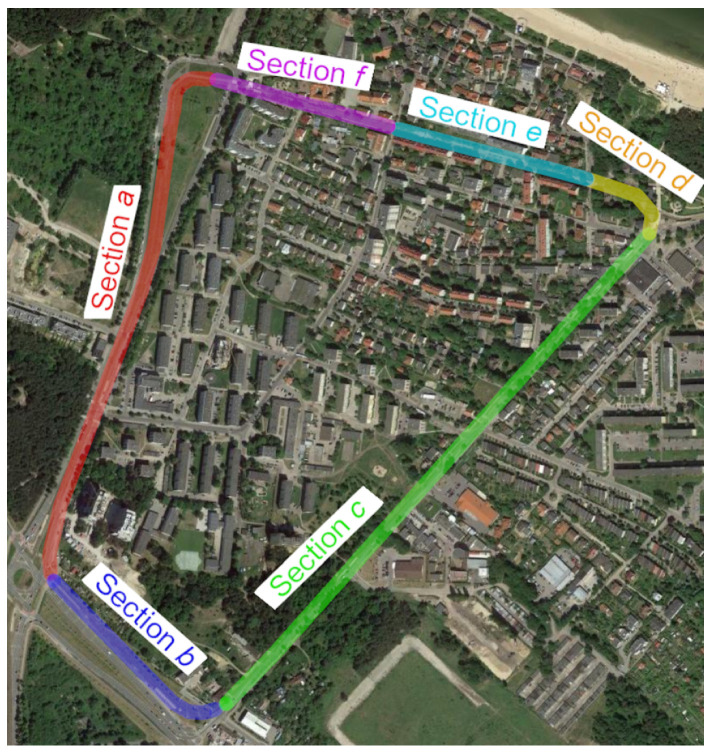
Measuring testing ground based on Google Maps.

The measurement campaign was carried out as a part of the project InnoSatTrack from 28.11.2018 until 29.11.2018 on a section of the tramway network in Gdańsk [22,24]. A large street terminus in Brzeźno district was selected (shown in Figure 1). The length of the test section was approximately 3 km, allowing multiple measurements of the same section overnight. Additionally, the system of the route in the form of a street terminus assured that there was no need to perform any maneuvers to prolong the duration of measurement sessions. Finally, the selected street terminus was characterized by diverse pavement conditions and the presence of field obstacles along particular sections.

**Figure 2:**
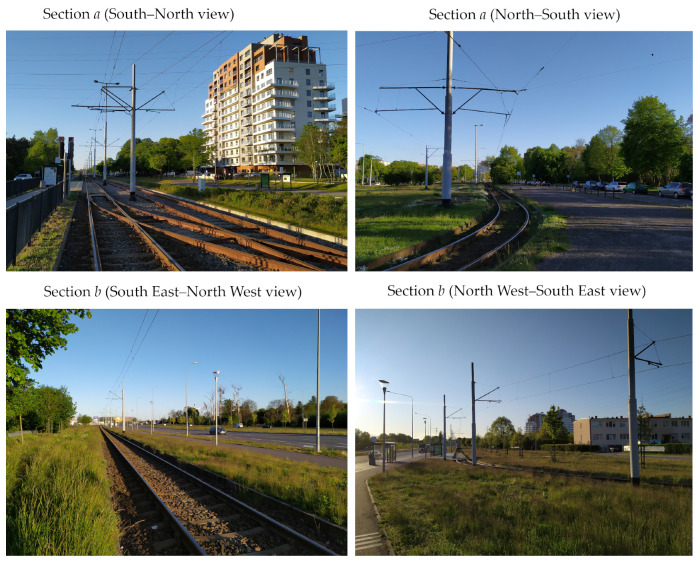
View of the streetcar track in the area of sections *a* and *b.*

**Figure 3:**
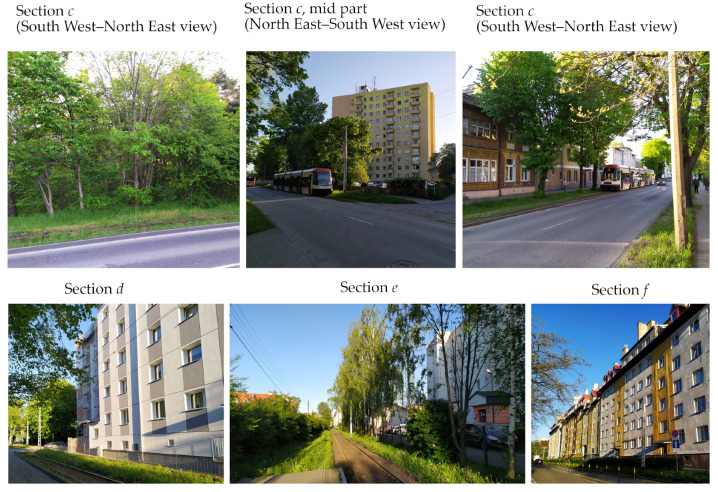
View of the tramway track in the area of sections *c*, *d*, *e* and *f.*

Detailed characteristics of sections of the selected terminus:

**Section *a***: Section of a length of about 850 m, starting at the terminus Brzeźno Plaża. Double-track tramway route is located on a strip dividing four-lane alleys. The section is characterized by a small number of field screens (single trees near the track at the terminus) and a very good technical condition of the track’s structure. The section was chosen as the reference section in relation to others.
**Section *b***: Section of a length of about 400 m. One-track tramway route is located asymmetrically on the north side of a four-lane avenue. The fragment is characterized by a small number of field screens (single trees near the track), and a very good technical condition of the track’s structure. This section was also chosen as a reference section.
**Section *c***: Section of a length of about 1000 m. One-track tramway route is located asymmetrically on the west side of a two-lane street. This section is characterized by a larger number of field screens than in previous sections (groups of trees and buildings located along the track) and very poor technical condition of the pavement (high values of twist and irregularities of the track in both the horizontal and vertical planes). This section was selected as the section to study the effect of track conditions on the accuracy of measurement.
**Section *d***: This section is the tramway route carried out independently of the road system with a length of approximately 150 m. This track is characterized by a complex geometry and the presence of the most extensive field screens (trees with crowns located over the streetcar track), and at the same time with a very good quality of the track. It was chosen to assess the impact of field screens on the possibility to reconstruct a complex geometric system of the track (field obstacles are shown in Figure 2). This section includes a diverging track to the Brzeźno terminus.
**Section *e***: Section of a length of about 330 m. One-track tramway route located asymmetrically on the south side of an access one-lane road. It is characterized by the appearance of very extensive field screens (tall buildings adjacent to the track) and the good condition of the track. It was chosen to assess the impact of field screens on the possibility of the reconstruction of a typical geometric system of the track (see Figure 3)
**Section *f***: This section is independent of the road system, and its length is about 270 m. It is characterized by an average number of field screens and good track condition. This section was chosen as a reference for section *c*.

Figure 2 shows the track in sections *a* and *b*, while Figure 3 shows examples of locations on sections *c*, *d*, *e* and *f*. Sections *a* and *b* are characterized by rather good conditions due to the visibility to satellites, while sections *c*–*f* are located in close proximity to high-rise buildings or trees. Here, disturbances in satellite signals from the sections of the northern edge of the terminus are expected.

### 2.2. Characteristics of Measurement Unit

To carry out the measurement, 10 GNSS receivers installed on a measurement platform were used. For the investigation, Leica GS18 and Trimble R10 receivers were selected. The corrections for real time kinematic measurement were obtained from the closest reference stations of the SmartNet and VRSNet networks for the Leica and Trimble receivers, respectively.

**Figure 4:**
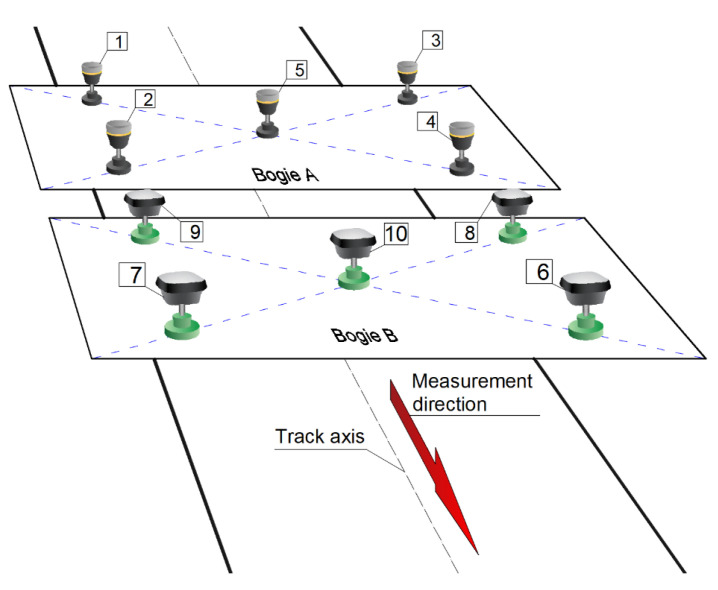
Diagram of the layout of GNSS receivers on the bogies.

As measurement cars, the bogies of the GWF 300 car series were adopted. They are characterized by favorable conditions for matching their ride axes into the track axis (no extensive amortization, no clearances) and a relatively low height of the measurement deck. On each of both bogies, five receivers were installed in a rectangular formation. In the centers of both bogies, directly above the bogie’s pivot, central antennas were placed. A diagram of the layout of the receivers is shown in Figure 4.

As the tramway bogies do not have their own drive device, there is a problem concerning the proper selection of a motor vehicle. The vehicle should meet the following requirements:

- The vehicle should provide both constant and low velocity of about 30 km/h. Only relatively modern tramways, equipped with power electronics drive control fulfill this requirement;
- The vehicle should provide a 230 V AC power supply to measuring devices and recording computers from the onboard network (preferably equipped with a standard 230 V socket).

**Figure 5:**
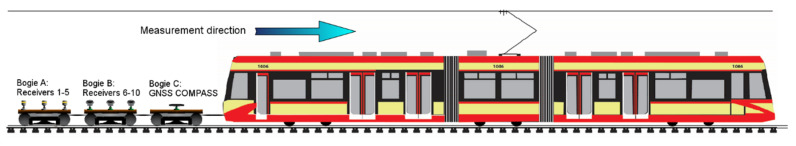
Diagram of the configuration of the measuring train.

The vehicle that met the above-mentioned requirements proved to be a Bombardier NGT-6/2. Finally, three measurement bogies were attached to the engine car. On the first bogie (the closest to tramway) the devices recording additional parameters of a run (such as acceleration, inclination and direction of the run) were installed. On the other two, a set of 10 GNSS receivers were placed. To avoid a negative influence from naturally occurring obstacles, i.e., tramway cabs, an additional bogie was located between the tramway and the bogies with GNSS antennas. A diagram of the measuring train is shown in Figure 5.

In the present measurement methodology, the fact is taken into account, that the position of the axis of the track relative to the rail vehicle is not unambiguously determined. One must keep in mind that each rail vehicle has a small degree of freedom in the lateral direction due to the bogie structural clearances and clearances between the rails and wheels. These clearances ensure the correct fitting of the bogie’s axles into the track. Moreover, besides of the constructional clearances of the bogies, displacements due to the spring amortization of the rolling stock occur. Taking into account also conicity in a rail and wheel interface, some kind of typical oscillation (so-called hunting oscillation) should be expected. However, this kind of movement is hard to assess without additional reference measurements (for example vision methods) and is not discussed in detail in this article.

**Figure 6:**
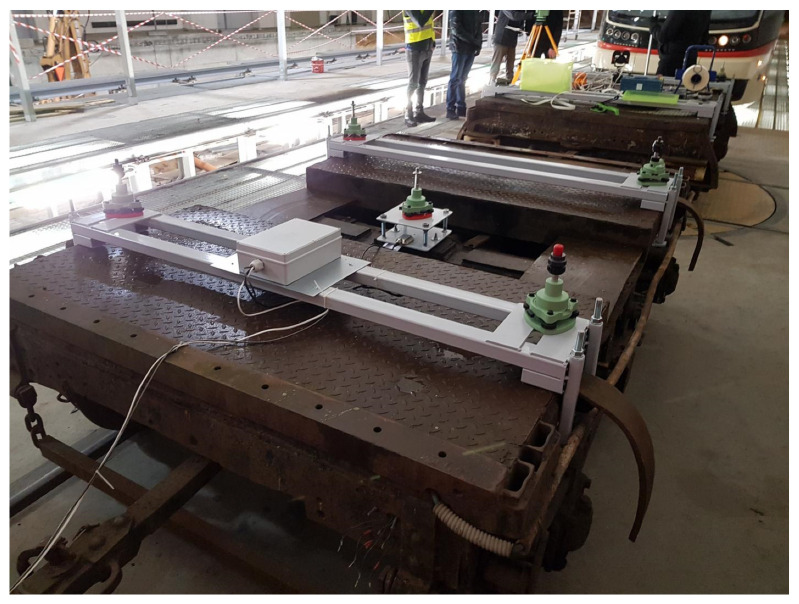
Setting out the position of receivers in relation to the bogie’s structure and the tramway track in the hall of the Wrzeszcz tram depot.

**Figure 7:**
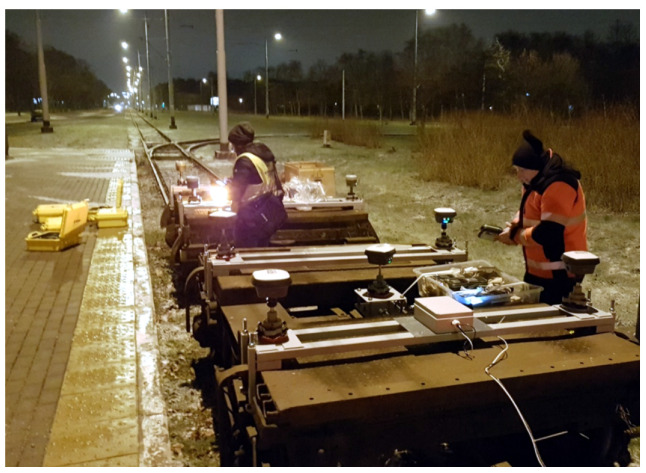
The measuring set used in the mobile satellite measurements duringmeasurements in Gdańsk.

It is therefore assumed, that the most reliable results with respect to the track axis are obtained by situating the antenna in the axis of symmetry with respect to the construction of the wheel set. This point for the construction of the bogie is in the pivot axis. This operation is difficult due to the limited access to the structural elements of pivot from the outside. Thus, there is the first factor related to the uncertainty of measurement, i.e., determination of the precise location of the base receiver on the vehicle’s platform. Due to the above problem, the positioning of the GNSS receivers’ centers was performed in conditions that minimize positioning errors. Installation of equipment was carried out in the hall of the tram depot. The process of measuring devices installation is shown in Figure 6. Special support structures enabled the precise installation of each tribrach according to the scheme assumed. Figure 7 presents the bogies instrumented with controllers and receivers during measurements.

During the measurement campaign, six passages on the specified street terminus were performed between 0:09 and 3:37. At this time, 10 receivers operated in continuous recording mode at a frequency of 20 Hz. During the study, a total of 1,186,518 positions were collected. All of the records met the assumed accuracy of designation (uncertainty radius not greater than 0.2 m).

### 2.3. Methodology for Determining the Main Directions of the Routes

**Figure 8:**
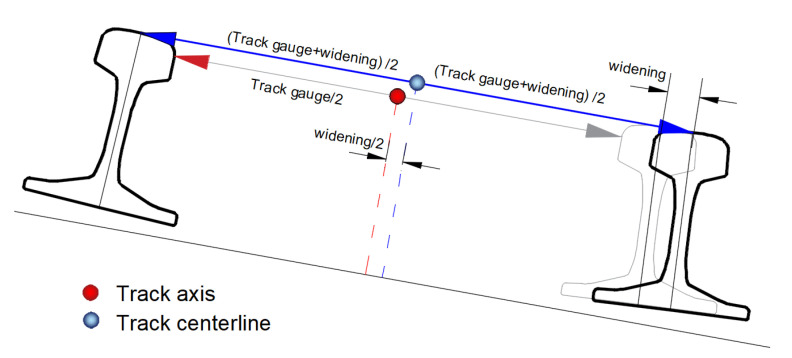
A schema of the location of the track axis and the track centerline.

The axis of the railway or tramway track physically does not have any representation. Considering a typical cross-section of a track, its axis is located at a point distant by a length of half the width (track gauge) between the rail courses at a height of the line connecting running surfaces of rail heads. In general, the axis of the track does not have to coincide with the central axis (so-called centerline) of a railway track. This is the case in curves with small radii, in which a widening of the track is often made. Figure 8 shows the definition of the axis of a railway track and its centerline.

Reconstruction of the track position in straight sections does not require additional adjustments related to the transverse or longitudinal inclinations. It is assumed that the track in the straight section is designed and built as a track without a lateral inclination (cant). Therefore, any found deformations resulting from the technical degradation of the track will be removed from the signal if necessary. Thus, the main directions will be assessed based on the analysis of the recorded positions on the straight sections of the track in particular passing measurements.

Prior to the analysis, the following assumptions were made:

- Multiple repeated measurement of a precisely indicated track section will enable to evaluate the error of the identification of the main directions. The directions will be defined on the grid of horizontal coordinates;
- Generally, the main directions will be designated by antennas positioned in the pivot axis of the measurement bogies;
- The geometric layout of the selected test section will be identified as a system of straight lines, regardless of the nature of deformation occurring in the track;
- Obvious deformations observed in the measurement signal, such as the so-called broken straights, will be removed, as they distort the assessment of the main directions;
- It was assumed that the assessment of the accuracy of the identification in the horizontal coordinate system will be made on the basis of the analysis of the reproduction of straight sections signals.

#### 2.3.1. Data

**Figure 9:**
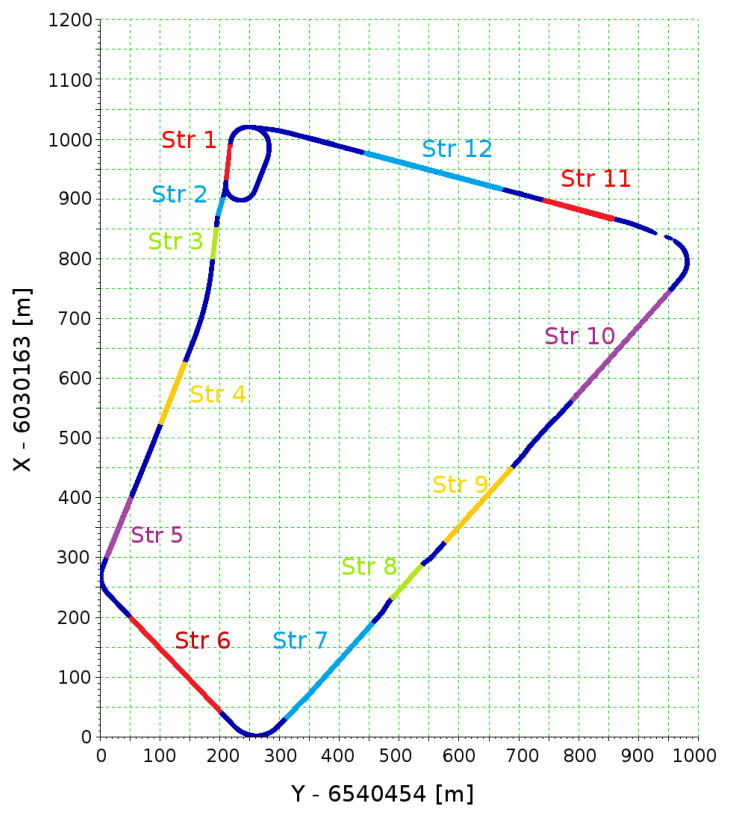
Measurement of the coordinates of the route in the PL-2000 system with the designation of individual straight sections.

The selected straights located in the designated sections of the tramway terminus were analyzed. Figure 9 shows the location of individual straights in the form of coordinates on the grid of the PL-2000 system in force in Poland (Gauss–Krüger projection for Geodetic Reference System ‘80). These straights were separated from GNSS measurement data based on the visual assessment of the results. Table 1 shows the signatures of straight lines in the corresponding sections of the tested track. Data in the form of coordinates and additional information, such as the accuracy recorded by the particular receiver as well as the number of satellites used to calculate the position at a given moment of time, were used in the analysis. Table 2 shows an example of the scope of measurement data.

#### 2.3.2. Main Directions

The main direction of the existing railway track is defined as a straight line that describes the rectilinear section of the track best or as a tangent to the reverse curves system at the point of their contact. The main directions form a system called a route’s polygon. 

As defined, the main direction is described by the parameters of a linear model equation of the straight line. In this analysis, these parameters were estimated by the least squares method. Suitable sets of points were selected for the analysis with YX coordinates in the PL-2000 system, which are the measured positions of GNSS receiver.

Signals from 10 receivers recorded during six measurement runs were analyzed. It can therefore be assumed, that each of the 60 signals is an independent description of the location of the analyzed section of track. Obviously, the most reliable signals are the signals recorded by antennas situated over the axis. In these places, transverse shifts of the tram bogies in relation to the track should be minimal. The antennas located at the corners of measuring platforms should also draw straight lines with the same direction coefficient. However, these positions can be subject to increased uncertainty due to the shifts and vibrations of the platform during test runs.

The signals recorded by individual antennas in each of the runs were analyzed in the local systems of coordinates, of which the abscissa axis is in accordance with the least squares line. This methodology allows for a clear interpretation of the differences between the signals recorded. A relatively large number of points used in the regression calculation makes it so that the straight lines are determined at a very low matching error, especially in situations of long straight lines. 

The methodology of transformation that uses homogeneous coordinates was accepted. The transformation of the YX coordinates of the PL-2000 system to homogeneous coordinates can be written as:

(1) $$\left(Y^{\prime },X^{\prime }\right)\to \left[\begin{array}{ccc} Y & X & 1 \end{array}\right]$$

Thus, transformations of coordinates can be described with a matrix equation in the form:

(2) $$\left[\begin{array}{c} \begin{array}{ccc} Y_{1}^{\prime } & X_{1}^{\prime } & 1\\ Y_{2}^{\prime } & X_{2}^{\prime } & 1\\ \cdots & \cdots & \cdots \end{array}\\ \begin{array}{ccc} Y_{n}^{\prime } & X_{n}^{\prime } & 1 \end{array} \end{array}\right]=\left[\begin{array}{c} \begin{array}{ccc} Y_{1} & X_{1} & 1\\ Y_{2} & X_{2} & 1\\ \cdots & \cdots & \cdots \end{array}\\ \begin{array}{ccc} Y_{n} & X_{n} & 1 \end{array} \end{array}\right]\cdot M$$

where M is a matrix of transformation in the form:

(3) $$M=\left[\begin{array}{c} \begin{array}{ccc} m_{11} & m_{12} & 1\\ m_{21} & m_{22} & 1\\ m_{31} & m_{32} & 1 \end{array} \end{array}\right]$$

The elements of matrix M ($m_{\mathrm{ij}}$, i=1,2,3, j=1,2) depend on a character of transformation and in specific cases of a single transformation this matrix takes the form of a translation matrix $T\left(t_{h},t_{v}\right)$, with rotation R(φ) or conversion of symmetry F performed with respect to the Ox or Oy axes of coordinates:

(4) $$T\left(t_{h},t_{v}\right)=\left[\begin{array}{c} \begin{array}{ccc} 1 & 0 & 0\\ 0 & 1 & 0\\ t_{h} & t_{v} & 1 \end{array} \end{array}\right];\;R\left(\varphi \right)=\left[\begin{array}{c} \begin{array}{ccc} \cos\varphi & \sin\left(\varphi \right) & 0\\ -\sin\left(\varphi \right) & \cos\varphi & 0\\ 0 & 0 & 1 \end{array} \end{array}\right];\;F=\left[\begin{array}{c} \begin{array}{ccc} \pm 1 & 0 & 0\\ 0 & \pm 1 & 0\\ 0 & 0 & 1 \end{array} \end{array}\right]$$

where:$\;t_{h},t_{v}$ are the distance from the origin to a translated point along the horizontal and vertical directions, and φ is the rotation angle of the vector of the transformed point with respect to the origin of the coordinate system.

### 2.4. Automatic Identification of the Polygon-Algorithm for Identifying Main Directions

The geometric layout of the railway in the horizontal plane consists of straight-line sections and the sections arranged in curves, consisting of circular arcs and transition curves with variable curvature along the length of the elements. The analysis presented in this paper concerns the polygon, which is formed by the main directions in accordance with the straight sections of the track or which are tangent to reverse curves. It is also assumed that two successive directions in places of their intersections are formed by the vertices of horizontal curves. The angle through which the direction changes from direction i to i+1 is the intersection angle.

For the description of polygon system, the equations of the straights of the YX coordinates (in accordance with the PL-2000 system) as $X=a_{md}\cdot Y+b_{md}$ was used. Parameters of the equation ($a_{md}$, $b_{md}$) refer to the main directions of the analyzed route.

The basic steps of the algorithm supporting the identification of main directions’ system are described below.

**Figure 10:**
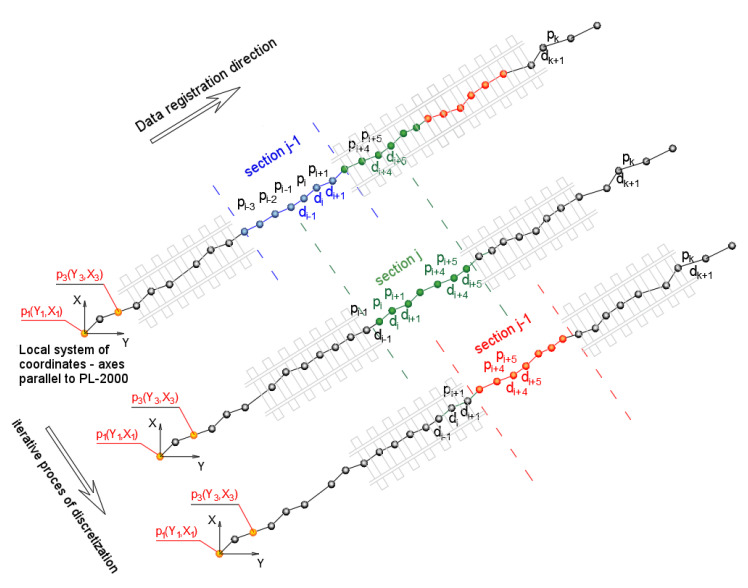
Schematic diagram of the route discretization into sections; $p_{i}$—measured points, $d_{i}$—distance between points.

(1) The first step is the discretization of the data to a finite number of sections. Fragmentation of the measurement signal took place on the basis of assigning each coordinate to intervals of a fixed length. At this stage the geometric system is not known, so the road is calculated by summing up distances from point to point. This is the first estimate of the distance along the route, which—when carried out in an appropriate way (filtering out concentrations of points recorded at standstills etc.)—will enable further analyses to identify the polygon. Figure 10 shows a schematic diagram of the route discretization, while Equation (5) shows a basic method of counting the distance along the route. The length of each section depends on the density of records and it is generally set in the phase of tuning the algorithm to the needs arising from the characteristics of the measurement signal.

(5) $$D_{i}=D_{i-1}+d_{i}=D_{i-1}+\sqrt{{\left(Y_{i+1}-Y_{i}\right)}^{2}+{\left(X_{i+1}-X_{i}\right)}^{2}}$$

where: $D_{i}$—distance from the starting point to the current one; $Y_{i},X_{i}$—flat coordinates of measuring points $p_{i}$ in the local system with directions in accordance with the PL-2000.

**Figure 11:**
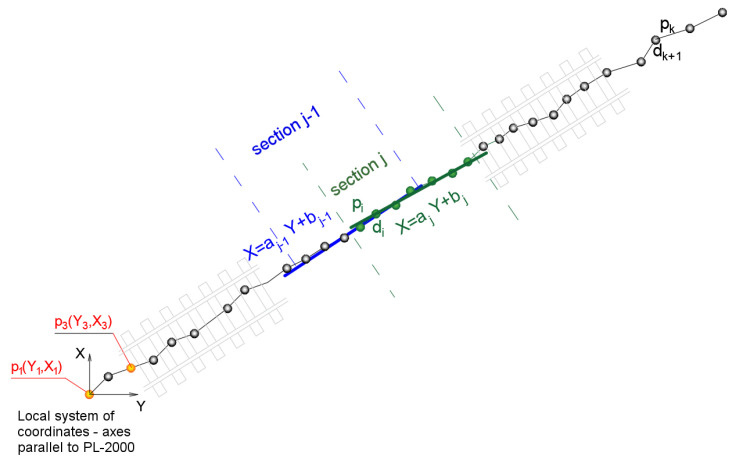
Schematic diagram of determining directions characterizing individual sections.

In each of the sections, there are a number of points with coordinates YX. When testing the algorithm, the minimum number of points in a particular interval was fixed; it should be remembered, that in continuous measurements there are places with no records of a measurement signal. Then, for each of the intervals the analysis of regression is made between the Y and X coordinates, due to which the estimate of the directions characterizing individual intervals of discretized route takes place, as shown in Figure 11. Due to the auxiliary character of estimated directions, the calculations were performed according to the least squares algorithm method for linear correlation. The method in this case involves the determination of the parameters of simple regression by minimizing the deviations of the measurement values of vertical coordinate X of the reference system PL-2000 (or the local system in accordance with the directions compatible with PL-2000) with respect to the linear model. The method accepted can be described with the equation:

(6) $$f\left(a_{j},b_{j}\right)=\overset{m}{\underset{i=1}{\sum }}{\left(X_{i}-\left(a_{j}\cdot Y_{i}+b_{j}\right)\right)}^{2}\Rightarrow min$$

where: $a_{j},b_{j}$—parameters of linear model in section j, and $Y_{i},X_{i}$—coordinates of measurement points $p_{i}$ in the local system with directions in accordance with the PL-2000.

Each section has its own estimator of direction, which can be the coefficient of the least squares straight slope or the angle to the axis of abscissae Y. It could also be the azimuth of the designated direction.

(2) With estimators of the direction in the individual sections of the route, variation of this direction is analyzed in the passages from one interval to another, i.e., in the series of consecutive sections. It is assumed that the angle representing the direction in each section maps the variation of the shape of the axis of the route analyzed. On the straight sections, a slight oscillation of the estimator value is expected, and in the areas of curves beside the oscillation a clear increasing trend of this value should appear. In order to examine these trends, the algorithm groups the adjacent sections with one another (of which each has a calculated estimator of direction in the previous step) in a minimum number and carries out the next analysis on the sets thus designated. The number of sections in each group depends on the geometric system (length of homogeneous sections of the route) and is determined by the analysis of Pearson’s correlation coefficient. This time, the analysis of regression concerns the values of the angles of individual sections. If we assume the angle as azimuth (Az) then the analysis of the grouped regressions of section can be written as follows:

(7) $$f\left(a_{g},b_{g}\right)=\underset{j}{\sum }{\left(Az_{j}-\left(a_{g}\cdot D_{mid_{j}}+b_{g}\right)\right)}^{2}\Rightarrow min$$

where:

$a_{g},b_{g}$—parameters of a linear model of the group of adjacent sections;
$Az_{j}$—angles characterizing the individual sections in the group;
$D_{mid_{j}}$—middle values of the distance covered in each section (equivalent to mileage).

**Figure 12:**
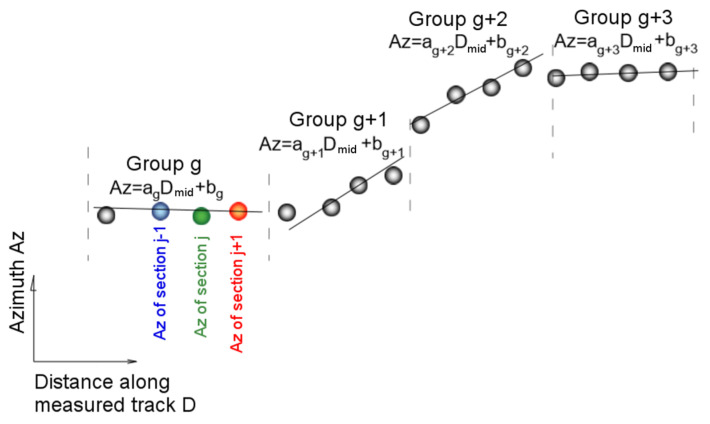
Schematic diagram of the analysis of the variability of azimuths along the discretized route.

Schematic diagram of the analysis of the regression of the sequence of angles indicated in the individual sections of discretized route is shown in Figure 12.

According to the assumptions, if the absolute value of the direction coefficient $a_{g}$ does not exceed the threshold value assumed in a particular group of sections, the algorithm assigns to a particular group (at this distance) the attribute of a straight section. The total length of the straight line is calculated as the sum of the lengths of the next (consecutive) groups of sections characterized by the stability of angle, and thus the direction coefficient of regression did not exceed a certain value close to zero. This approach makes it possible to find the main direction as well in combination with reverse curves. Then, the location with the value of regression close to zero will appear in the analysis.

**Figure 13:**
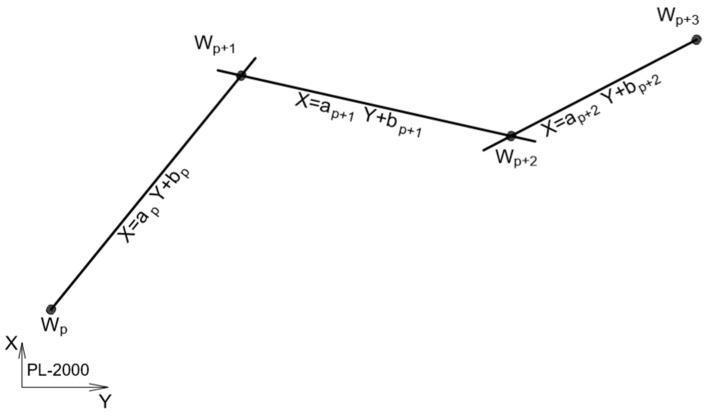
Schematic diagram of the reconstructed vertices system.

(3) Once the records belonging to the straight sections are identified, the total, i.e., the linear regression defined on the whole set of points, is determined. Due to the fact that the straight sections can vary in length and for longer sections precision parameters of determining the position can undergo dynamic changes, the authors used a weighted regression analysis, in which the weights are defined by measurement uncertainties recorded for flat coordinates by GNSS receivers. The method implemented in this way maps the main directions on the sets of points with the differentiated distribution of position error more accurately than the simple linear regression (unweighted).

(4) The coefficients of the equations of straights constitute the basis for building the polygon, in which the coordinates of vertices are calculated as points of the intersection of adjacent (by indexation) straight lines. The coordinates of the points of the start and end points of the polygon are calculated based on measured extreme coordinates. A schematic diagram of the reconstructed system of vertices is shown in Figure 13.

(5) The last step of algorithm is the analysis of the slopes of the adjacent straight lines. In this step, it is possible to merge the adjacent straight lines with one another. This is the moment in which the two adjacent straight lines with different direction slopes should be accepted (or not) as the area of the change of main directions. The adjacent two straight lines can therefore be designated as two tangents of a horizontal curve or connected together in one tangent (main direction) of the polygon.

## 3. Results

For detailed analyses, the data obtained from receivers number 5 and 10 were selected. These receivers were mounted on the pivots of the tramway bogies, which means that the trajectory recorded by them in the horizontal plane maps the axis of the track. The exceptions are sections located in the curves with a cant, but the test section was selected in such a way that the impact of the cant is as small as possible. These receivers recorded a total number of 253,435 coordinates during the passage through the test section.

**Figure 14:**
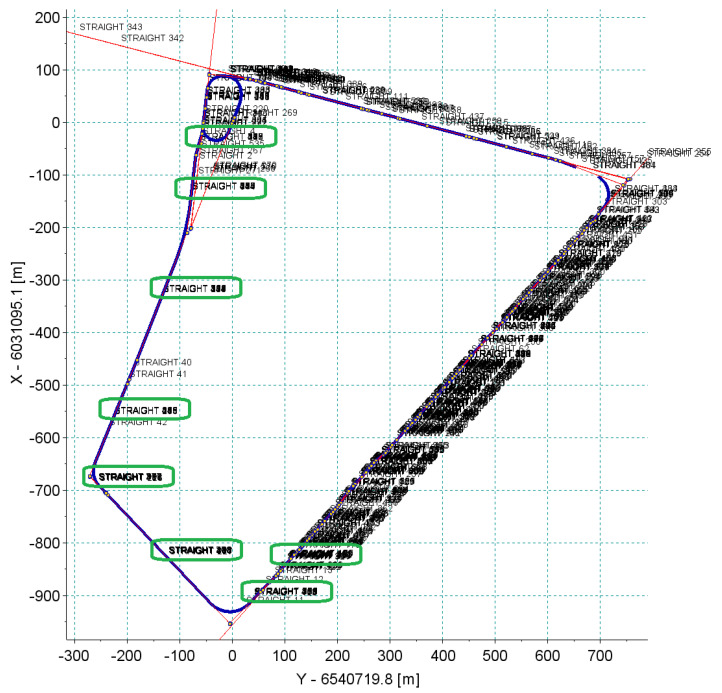
Automatic division of the measuring route into straight sections.

**Figure 15:**
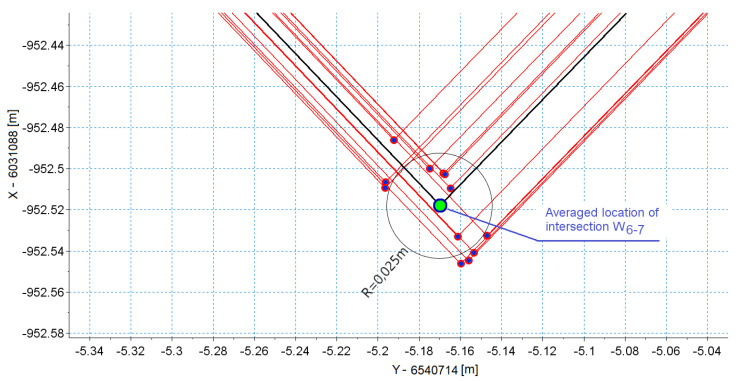
Result of the multiple reconstruction of the selected vertex (intersection) of the polygon.

**Figure 16:**
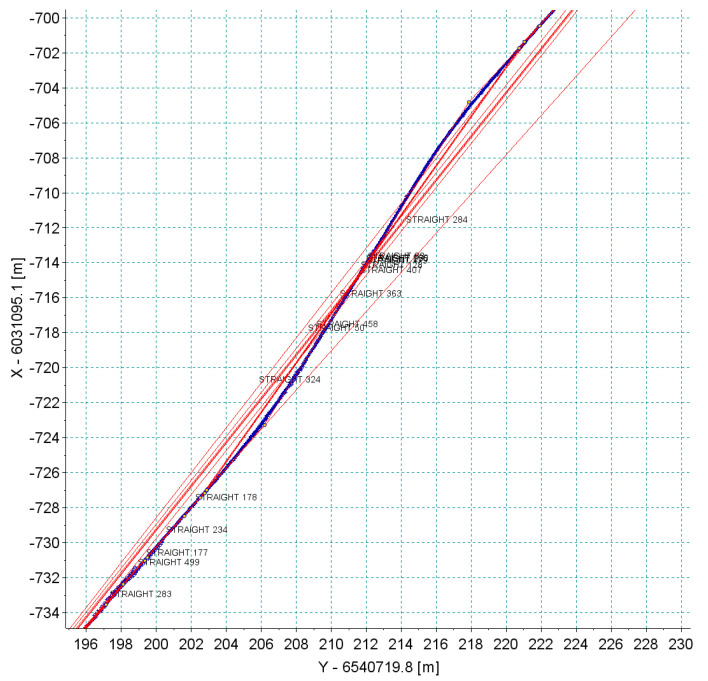
Close-up of the system with reverse curves.

Then, the analysis of the resulting measurement signal was made, according to the algorithm shown in Section 2.4. For input data in the form of the measurement signal recorded by two receivers (with a frequency of 20 Hz) during the six passages of the route, the algorithm detected the presence of 536 straight sections, giving an average of about 45 straight sections making up the whole geometrical system. Figure 14, Figure 15 and Figure 16 show the calculation results of the algorithm. They represent scalable windows of a program for data analysis, implemented in a Scilab environment. The automatically developed computer program defined the beginnings and ends of the straight sections, and in half the reconstructed section a label with the next number of section was pasted. Since the algorithm operated simultaneously on all 12 measuring signals, these fragments, which a number of straight lines overlap, can be treated—as the way of visual assessment of the result in the graph—as repeatedly reconstructed. If the numbers of straight lines are offset with respect to one another, it means that the algorithm was not able to reconstruct the beginnings and ends of straight or divided sections into shorter ones at a high level of repeatability, or indicated that the straight lines as false positive, recognizing, for example, the distortion of a signal or inequality of a track in a straight section as a curvilinear section.

It can be noted that on sections *a* and *b*, the algorithm identified the vertices most accurately. Figure 15 shows the vertices determined on the basis of identified straight lines in sections *b* and *c*. Section *c* compared to section *b* featured less precise characteristics of the straight lines reconstructed. The result was a clear dispersion of the designations of vertex in the whole series of measurements. In addition, Figure 14 shows that on the other sections of the test track (mostly *c*, *d*, *e* and *f*) a series of very short straight sections were measured, hindering the overall analysis (expressed as a loss of clarity of description at the scale assumed). In addition, in section *c* there were fragments of the track with very short reverse curves (shown in Figure 16), which constituted an additional problem for the automatic identification of the algorithm.

## 4. Discussion

### 4.1. Analysis of Main Directions

Bearing in mind that in the case of tramway lines, deformations of the track axis in the horizontal plane can be of similar length to the short straight sections—full automation turned out to be very difficult. In this situation, for the further analysis of the main directions only those sections were selected for which there was little doubt that they constitute the main directions of the route.

**Figure 17:**
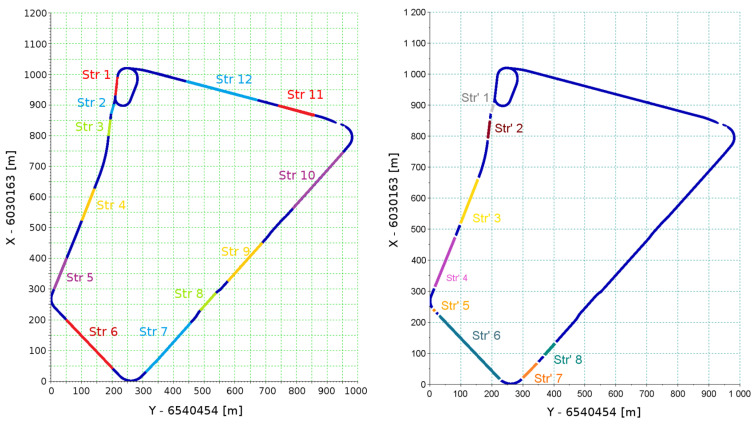
Comparison of the set of selected straights through a visual assessment (**left**) to a set of straights identified automatically (**right**).

Among the sections identified by the algorithm as straight sections, *a*, *b* and *c* were selected for further analysis of eight straight of sections. Figure 17 shows the final selected straights and straights selected initially (on the basis of a visual evaluation of the measurement data). To distinguish the straight lines from the earlier selected sections (Figure 9), for the first ones *Str’* markings were introduced. 

Comparing both sets of straight lines, it can be concluded that there was a large convergence of both methods. There were significant differences in the division of *Str 6* to *Str’ 5* and *Str’ 6* and division of *Str 7* to *Str’ 7* and *Str’ 8*. It is worth noting that the reconstructions of straight sections of relatively short length and the longest ones were different. In addition, existing GNSS measurement uncertainties affect the repeatability of the reconstruction of very short sections. However, the uncertainty of designating the angle in the case of short sections will cause small deviations of the model course of the straights with respect to the actual course. However, in the case of long sections, even a small error in the designation of angle can have a significant effect on the results obtained at the ends of the reproduced geometric layout. That is why the indicator taking into account the length of a section was introduced. This indicator, designated as $\sigma_{Az\left(rel\right)}$, is the standard deviation of the reconstructed azimuth multiplied by the length of section. The obtained parameters of the sections examined are shown in Table 3 and Table 4.

For each measurement, a set of coordinates was determined. Then, the mean and standard deviation of the corresponding values were calculated. Based on the coordinates of vertices the distances |∆W| between these vertices were calculated (of the individual measurements i=1,…,n) as well as the averaged vertex $X_{av},\;Y_{av}$. The measure of uncertainty determined the vertex based on the straights reconstructed were therefore the distance ${\left|∆W\right|}_{av}$ and its standard deviation $\sigma_{\left|∆W\right|}$. All parameters describing the vertices, together with intersection angles α, are shown in Table 4.

The values in Table 1 and Table 2 clearly show that Section 5 and the associated vertex W5–6 were reproduced with the highest uncertainty. Section 5 is a straight insert between the crossover needle (the measurement train moved in the reverse crossover direction) and the curve behind the crossover. This section is very short, and the intersection angle at this point is very small (less than 1 degree). The measurement on such a short section is burdened with considerable uncertainty to designate its direction, and also coordinates of vertices W4–5 and W5–6. Additionally, the vertices analyzed featured by a very small intersection angle, comparable to the value with the uncertainty to determine the angle. The coincidence of a short length of Section 5 and the small intersection angle in vertex W5–6 generated a very high level of uncertainty (mean value of error and standard deviation at 1.3 m). However, this was not the result of an incorrectly accepted measurement technique, but of geometric relationships in the measured geometric system. The coordinates of vertices with the favorable system of tangents were determined with very high reproducibility.

### 4.2. Accuracy of Measurement Based on the Repeatability of Signals

**Figure 18:**
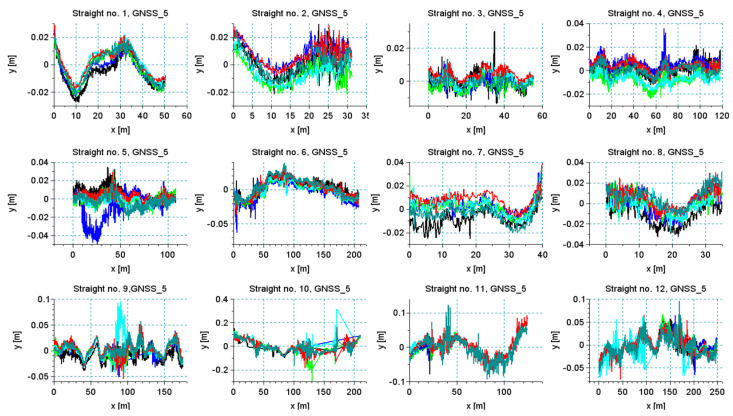
The results of a series of six measuring sections *Str 1*–*Str 12* recorded by receiver no. 5.

**Figure 19:**
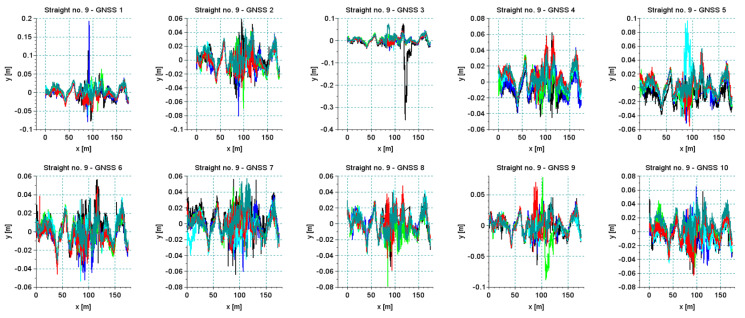
The results of a series of six measuring section of *Str 9* characterized by poor track conditions, recorded by all receivers of the measurement system.

All originally selected straight sections were analyzed. The existence of numerous unfavorable deformations of the track and the field conditions for GNSS measurement should appear in the analysis of signal reproducibility. Figure 18 shows the results of all measurement series—for each straight section—recorded by one of the antennas positioned on the pivot (receiver 5). In most cases, a very good repeatability of the trajectory of this device was observed. It is clear that in cases of bends on the straight lines all runs recorded the same qualitative character of deformation. This observation makes it possible to trim signals so that they do not distort the precision assessment. Of course, cutting out only the deformed parts from the straight line disturbs the actual direction of the straight line; however, to assess the accuracy of the measurement itself such an operation is necessary. Figure 19 shows the results recorded by all receivers during the measurements of section *Str 9*. This section features the poor track conditions. Numerous irregularities and deformations caused the occurrence of vibrations on the measurement vehicle. Graphs of recorded signals indicated a large number of bends (deformations) along the section of this track. This is one of the sections where the algorithm of the detection of main directions selected a series of short straights. The signals recorded by all receivers revealed the same deformation of the track.

**Figure 20:**
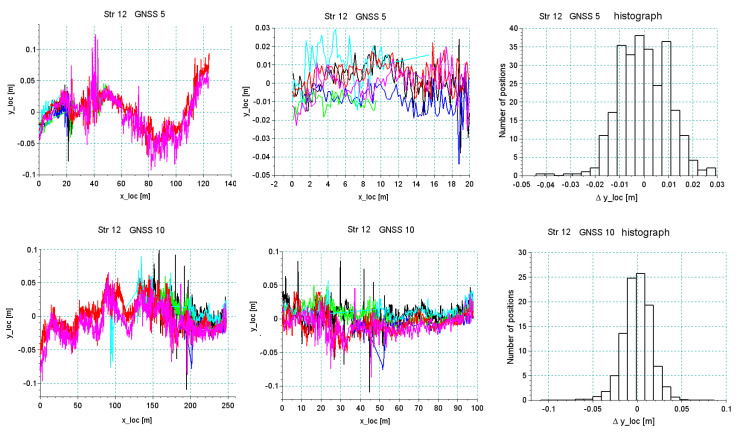
Analysis of the accuracy of the reproduction of the main direction in section *Str 12* featuring extremely unfavorable field screens.

**Figure 21:**
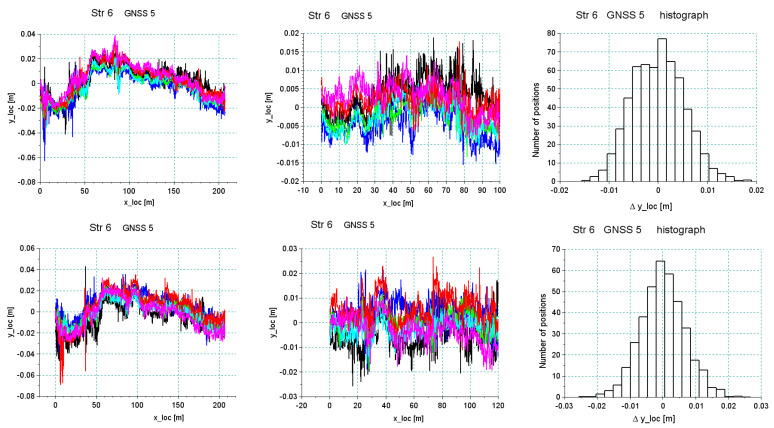
Analysis of the accuracy of the reproduction of the main direction in section *Str 6* featuring very good visibility of satellites.

Figure 20 and Figure 21 show the results of the analysis of the reproducibility of the data recorded by receivers 5 and 10 for sections *Str 12* and *Str 6*. As seen in Table 1, these sections were characterized by a very unfavorable environment in the form of buildings and trees. This resulted in a discontinuity in data recording during part of the particular run. The analysis was done for a short segment that was recorded during all six crossings. As regards the accuracy in the reconstruction of direction, the standard deviation was at a level of 1–2 cm, depending on the recorded segment of a particular section, but reliability of measurement under these conditions was relatively low—every crossing showed disturbances of signal loss by different receivers.

The example of section *Str 6* (Figure 21) shows that in the accepted methodology of measurement, a very high reproducibility of independent measurements can be achieved, which directly proves a high precision of the reconstruction of the main directions of the tested route. However, the overall assessment ultimately depends on the field conditions in which the measurement is made. Systems based on several receivers operating independently increase the reliability. Therefore, even in difficult measurement conditions, data collection is still possible. However, the scope can be limited to certain sections of the route.

**Figure 22:**
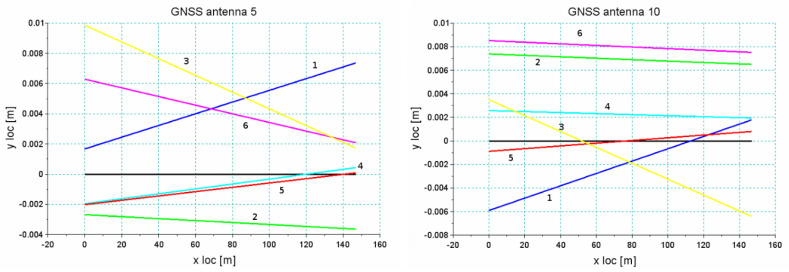
The relative position of straight line *Str 6* estimated from individual runs around the least squares straight line—antenna no. 5 (**left**) and no. 10 (**right**).

Figure 22 shows a graph of least squares lines in the local coordinate system for section *Str 6*. Also in this case, reference straights were brought to the local system, which facilitated the assessment of mutual relationships with individual straights. The black line represents the estimator of the expected value, that is, the real position of the track (reduced to level 0 of the local system). The lines in other colors are six estimated straight lines from the six individual runs. The graphs show that the maximum values of the straight lines most distant from one another did not exceed 1 cm. 

## 5. Conclusions

The selected test track met the assumptions as regards different conditions of satellite measurements. The analysis of the results clearly showed the impact of urban infrastructure on the possibilities of the reproduction of main directions along tramway lines. The algorithm presented using the regression analysis of measured points (representing the track axis) with the appropriate approach allowed efficient determination of the position of straight sections in a global system of references. However, the specification of geometric systems of tramway tracks (short geometric elements) led to the analysis of the main directions requiring a sufficiently high density of data. In the case of a large number of points, which are burdened with a high level of uncertainty, there is a risk of incorrect estimation of the identified parameters of the route directions, which in turn leads to the incorrect reconstruction of the polygon. The biggest problems with the polygon reconstruction are the intersection angles with very short tangents. The occurrence of small values of intersection angles (about 3 degrees or less) generates a relatively large uncertainty of the reproduction of the intersection of tangents (vertices). Finally, the coincidence of small intersection angles with short tangents makes that even a small deviation of the direction of the tangent causes very significant differences in the coordinates of the polygon’s vertices.

As shown, the tramway infrastructure is characterized by an extremely unfavorable shape from the point of view of the reproduction of geometric systems in the global system. It is dominated by very short-length straight sections and curves with a complex system. In the measurements made, the shortest uniquely located straight section had a length of 0.7 m. It was on the extension of the main track in the crossing (straight section inserted within the turnout construction). Since railway systems have much longer straight sections, a successful measurement made on the tramway tracks is a positive prognostic for further research related essentially to the inventory of railway lines.

The analysis also showed that the geometric condition of track (rails’ deformations and geometric imperfections) has a significant impact on the identification of the main directions of the route. One of the straight lines in the street terminus analyzed featured a high degree of geometric deformation, with the result that the algorithm of the identification of the main directions did not distinguish real directions (design) from the short straight lines resulting from the deformations present. The automatic detection of straight sections in such conditions turned out to be very difficult, requiring more advanced computational algorithms. Sections characterized by a good geometric condition were identified properly and the polygon designated on this basis was characterized by a relatively small error of the reproduction of vertices, at the level of a few centimeters.

## Figures and Tables

**Table 1:** Selected straights in the scope of the *a*–*f* sections of the geometric layout analyzed.

Straight Line	Location in the Route	Assessment of Track Condition/Quality	Assessment of Visibility
Str 1	Sect. a	Fair	Good
Str 2	Sect. a	Excellent	Fair
Str 3	Sect. a	Excellent	Good
Str 4	Sect. a	Excellent	Excellent
Str 5	Sect. a	Excellent	Good
Str 6	Sect. b	Excellent	Excellent
Str 7	Sect. c	Bad	Poor
Str 8	Sect. c	Bad	Poor
Str 9	Sect. c	Bad	Fair
Str 10	Sect. c	Bad	Poor
Str 11	Sect. d	Good	Bad
Str 12	Sect. e	Good	Poor

**Table 2:** Input data for the analysis of the main directions for a selected straight.

Record No.	GPS Time (s)	Horizontal Coordinate Y (m)	Vertical Coordinate X (m)	Two Dimensional Position Error (m)	Number of Satellites
358670	2,029,347,009.00	6,540,504.8138	6,030,362.9699	0.0400	8
358671	2,029,347,009.05	6,540,504.9345	6,030,362.8442	0.0400	8
358672	2,029,347,009.10	6,540,505.0561	6,030,362.7132	0.0400	8
358673	2,029,347,009.15	6,540,505.1887	6,030,362.5797	0.0400	8

**Table 3:** Comparison of the reconstructed parameters of straight sections.

Section Number	Number of Measurements n	$\mathrm{Average}\;\mathrm{Length}\;L_{av}\;[m]$	$\mathrm{Average}\;\mathrm{Azimuth}\;Az_{av}\;[\deg]$	$\mathrm{Standard}\;\mathrm{Deviation}\;\sigma_{Az}\;[\deg]$	$\mathrm{Indicator}\;\sigma_{Az\left(rel\right)}\;[\deg*m]$
1	12	19.1	196.139	0.026	0.500
2	12	71.5	186.536	0.002	0.116
3	11	194.9	201.427	0.001	0.176
4	12	187.1	202.196	0.002	0.332
5	12	0.7	135.792	0.346	0.233
6	12	274.5	136.415	0.002	0.670
7	12	50.9	44.051	0.012	0.603
8	7	37.3	41.706	0.008	0.313

**Table 4:** Comparison of the reconstructed parameters of the polygon’s vertices.

Marking of Vertex	$\mathrm{Angle}\;\mathrm{of}\;\mathrm{Return}\;\alpha_{av}\;[o]$	Number of Measurements n	$\mathrm{Vertical}\;\mathrm{Coordinate}\;X_{av}\;[m]$	$\mathrm{Standard}\;\mathrm{Deviation}\sigma_{X}\;[m]$	$\mathrm{Horizontal}\;\mathrm{Coordinate}Y_{av}\;[m]$	$\mathrm{Standard}\;\mathrm{Deviation}\;\sigma_{Y}\;[m]$	$\mathrm{Distance}{\left|∆W\right|}_{av}\;[m]$	$\mathrm{Standard}\;\mathrm{Deviation}\;\sigma_{\left|∆W\right|}\;[m]$
W1-2	9.603	12	−69.656	0.011	−62.422	0.064	0.049	0.041
W2-3	14.891	11	−86.535	0.007	−209.746	0.025	0.023	0.011
W3-4	0.769	11	−181.861	0.152	−452.648	0.391	0.337	0.226
W4-5	66.404	12	−271.796	0.098	−673.074	0.247	0.150	0.215
W5-6	0.623	12	−240.944	1.284	−704.802	1.339	1.261	1.306
W6-7	92.364	12	−5.170	0.017	−952.518	0.020	0.024	0.008
W7-8	2.345	7	86.052	0.126	−858.219	0.125	0.137	0.099
